# Correction to “Combined inhibition of de novo pyrimidine synthesis and ATR promotes ATF4‐mediated cell death in *TP53*‐deficient ALL”

**DOI:** 10.1002/hem3.70502

**Published:** 2026-10-05

Grünewald KJT, Kes MMG, Stoll GR, et al. Combined inhibition of de novo pyrimidine synthesis and ATR promotes ATF4‐mediated cell death in TP53‐deficient ALL. *HemaSphere*. 2026;10(7):e70439. doi:10.1002/hem3.70439

In Figure 5, the abbreviation ATR was incorrectly defined as “Activating Transcription Factor 4” in the caption. The correct definition is “Ataxia Telangiectasia and Rad3‐related (ATR) protein.”

We apologize for this error. The article has been corrected.

